# Precision oncology- the future of personalized cancer medicine?

**DOI:** 10.1038/s41698-017-0010-5

**Published:** 2017-03-20

**Authors:** Ann M. Bode, Zigang Dong

**Affiliations:** The Hormel Institute, University of Minnesota, Austin, MN USA

You’re living the good life—eating right, exercising, maintaining a healthy weight—doing all the recommended things to maintain a healthy and productive lifestyle. Then, Bam! Your health care provider convinces you that you should do a screening test for cancer during a minor surgical procedure. You protest because you feel great—never better—but you finally agree. You take the test, fully expecting to walk out with totally negative results and a clean bill of health. Then the hammer falls—the doctor bluntly says, “You have a tumor and you need to schedule surgery as soon as possible to remove the tumor and part of the affected organ”. This is surreal—you have no symptoms and you feel great. The surgery goes smoothly and you go home in only 2 days, a record for this kind of surgery. You don’t even have any real pain. The doc says all went extremely well, and the tumor appears to be contained but the pathology results are necessary to confirm the success of the procedure. Then you receive another unexpected blow—the microscopic examination says the tumor has invaded the lymph nodes and you will have to have chemotherapy. You feel like your world has turned upside down and is spinning out of control. How and why could this possibly happen?

Epidemiology suggests that cancer occurs more in men than women, in older vs. younger people, smokers vs. nonsmokers, blacks compared to whites, obese rather than thin individuals, sedentary vs. active people, those who consume an unhealthy diet compared to those who eat healthy, and in individuals with a low compared to a high economic status. However, the reality is that cancer can attack and invade anyone of any color, ethnicity, race, gender, or sexual orientation. We continually search for answers as to why cancer is so universal yet unique from case-to-case. Cancer is a multitude of different diseases, and shows no bias. One size does not fit all for cancer prevention or treatment. Even though the collective human genomes are more similar (>99%) than different (<1%), these small differences can have major widespread consequences. Research results over the last few years have shown that individual genetic differences account for many discordances in cancer susceptibility or resistance. Genome and epigenome-wide, transcriptomic and proteogenomic profiling techniques are now available that facilitate the detailed characterization of cancer at the level of the individual, which offers new opportunities for actual tailored approaches for treatment and patient care.

We are entering an era of rapidly evolving transformation in cancer research as it relates to medical practice, and a shifting paradigm of standardized health care in which detailed genetic and molecular information regarding a patient’s cancer is being used for personalized precision treatments. This transfiguration is supported by funding increases to advance the field of precision medicine under President Obama’s Precision Medicine Initiative and the White House Cancer Moonshot Task Force chaired by Vice President Biden. The Moonshot is charged with accelerating the pace of cancer research, and the advances that research brings to cancer patients in the U.S. and the global community. Additionally, the National Institutes of Health launched its own Precision Medicine initiative in 2015.

Sequencing of individual patient tumors has revolutionized oncology because the genetic information facilitates the elucidation of molecular changes in a specific patient’s cancer. What this genetic information has provided is the understanding that not only do tumors differ within a sample (tumor heterogeneity), but the information clearly shows that each patient’s individual tumor is an assemblage of inimitable molecular irregularities that will require an equally exclusive personalized therapeutic regimen.

npj Precision Oncology is an international, peer-reviewed journal committed to publishing cutting edge scientific research in all aspects of precision oncology from basic science to translational applications to clinical medicine.

Our goal for npj Precision Oncology is to provide a forum for discussion of advances at all levels that contribute to treating and preventing cancer. The journal defines precision oncology as “cancer diagnosis, prognosis, prevention, and/or treatment tailored specifically to the individual patient based on the genetic profile of the patient.” High-impact articles that entail relevant studies using panomics, molecular, cellular, and/or targeted approaches in the cancer research field are considered for publication. Papers will encompass all the areas of precision oncology from basic research to clinical applications.

Featured topics of the journal include targeted immunotherapy, mechanism-based therapies aimed at specific cellular signaling pathways, carcinogenesis including genetic risk factors, drug resistance mechanisms, reoccurrence, micro-RNA, tumor microenvironment, cancer metabolism, cancer stem cells, intervention strategies, emerging clinical and translational aspects and noncoding RNAs, including pseudogenes, circRNAs, and long noncoding RNAs. Papers are also encouraged that focus on the study of the relationship between inter-individual genetic variation and drug responses (pharmacogenetics), the visualization of functional behavior of tumor cells (molecular imaging) and its potential role in daily clinical practice.

The journal is committed to providing an efficient publishing service to the community. An expert Editorial Advisory Board, independent editors and a streamlined peer-review system enable rapid and fair decision making through to publication. The final manuscripts will also be enhanced by the editorial process making them attractive and accessible to the broad readership of the journal.

The journal publishes original basic science, translational and clinical research articles, case reports, brief communications, commentaries, perspectives, editorial summaries, meeting reports and review articles. To encourage communication between disciplines, the journal also will publish a professionally written Editorial Summary to accompany each research article, which summarizes the key issues being addressed within the article aimed to keep readership at the forefront of new discoveries in the field. This will make findings more accessible and understandable to a wide range of scientists, physicians, patient advocates, policy makers and patients themselves. The summaries will attempt to simplify and present even the most complex research papers in a user-friendly format for all readers, particularly patients. The journal will therefore serve not only the scientific community but also, equally essentially, people with cancer and their care partners—empowering them with access to information that is highly relevant to the struggle of living with and surviving cancer. We will highlight work from leading researchers related to key advances in basic science, genetics, and as well as clinical trials of therapies, interventions, and multidisciplinary care in cancer.

